# Supplementary material for: A pan-cancer analysis of homeobox family: expression characteristics and latent significance in prognosis and immune microenvironment
Source: Front Oncol. 2025 Feb 6;15:1521652. doi: 10.3389/fonc.2025.1521652 (PMC11840236; doi:10.3389/fonc.2025.1521652)
Supplement: Supplementary Figure 1 — Mutations of each HOX gene in 32 cancers (TCGA, PanCancer Atlas) by cBioportal. [file DataSheet1.zip › Suppl.files/raw code.zip!/code/2.expression/differential expr analysis/HOXA6.diff.pdf]

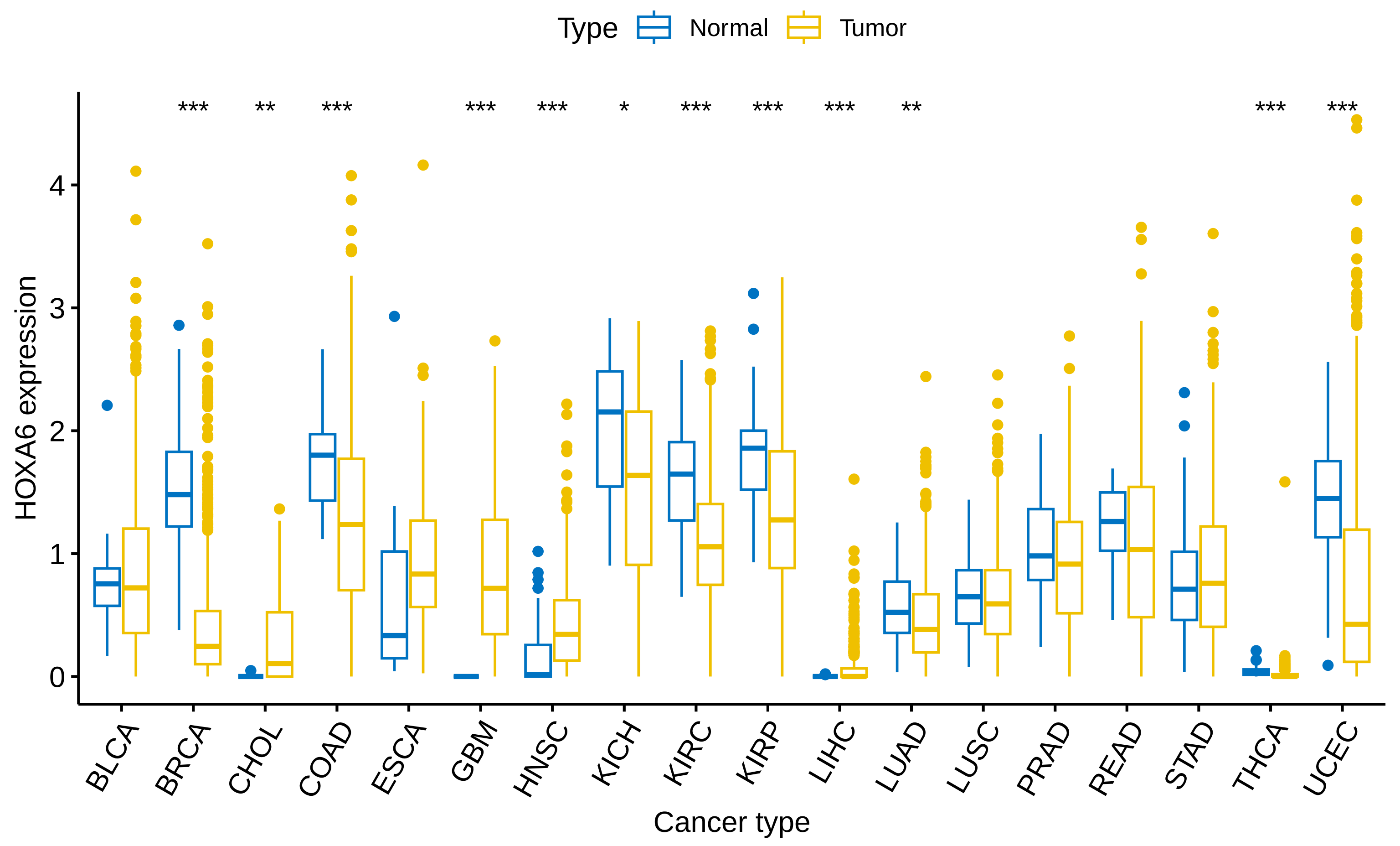

Type
Normal
Tumor
HOXA6 expression
0
1
2
3
4
***
**
***
***
***
*
***
***
***
**
***
***
BLCA
BRCA
CHOL
COAD
ESCA
GBM
HNSC
KICH
KIRC
KIRP
LIHC
LUAD
LUSC
PRAD
READ
STAD
THCA
UCEC
Cancer type
